# Pediatric Cardio-Oncology: Screening, Risk Stratification, and Prevention of Cardiotoxicity Associated with Anthracyclines

**DOI:** 10.3390/children11070884

**Published:** 2024-07-22

**Authors:** Xiaomeng Liu, Shuping Ge, Aijun Zhang

**Affiliations:** 1 Department of Pediatrics, Qilu Hospital of Shandong University, Jinan 250012, China; 2 Department of Pediatric and Adult Congenital Cardiology, Geisinger Clinic, Danville, PA 17822, USA

**Keywords:** pediatric, cardiotoxicity, anthracycline, risk stratification, monitoring, prevention, childhood cancer survivors, cardio-oncology

## Abstract

Anthracyclines have significantly improved the survival of children with malignant tumors, but the associated cardiotoxicity, an effect now under the purview of pediatric cardio-oncology, due to its cumulative and irreversible effects on the heart, limits their clinical application. A systematic screening and risk stratification approach provides the opportunity for early identification and intervention to mitigate, reverse, or prevent myocardial injury, remodeling, and dysfunction associated with anthracyclines. This review summarizes the risk factors, surveillance indexes, and preventive strategies of anthracycline-related cardiotoxicity to improve the safety and efficacy of anthracyclines.

## 1. Introduction

Anthracyclines refer to various glycosides with a chemical structure of 7, 8, 9, 10-tetrahydrocenequinone-5, 12, which is a chemical produced by microorganisms with antitumor activity. Such drugs include doxorubicin, epirubicin, pyranorubicin, daunorubicin, mitoxantrone, alorubicin, and idarubicin, among others. Of these, daunorubicin is the first synthetic anthracycline used for clinical treatment that is still suitable for the treatment of leukemia: It has contributed to the increase in the 5-year survival rate in children with leukemia from 30% in the 1960s to more than 90% at the time of writing [1]. Subsequently, the advent and application of other anthracyclines have greatly improved the long-term survival rate of patients with solid tumors [2]. However, anthracyclines induce severe cardiotoxicity, which not only limits the clinical use of anthracyclines but also affects the quality of life of children with cancer as a result of irreversibility and the dose response. With the improvement in anticancer treatment programs and the growing number of long-term survivors, the mortality rate from complications such as cardiovascular disease has exceeded the recurrence factor and has become an important factor affecting the survival of children with cancer [3]. At present, carrying out relevant research to explore effective predictors and intervene early has become a top priority. The task of maximizing the long-term quality of life and survival rate of childhood cancer survivors is an important one in pediatric cardio-oncology [4]. We reviewed previously published articles to analyze and explore anthracycline-related cardiotoxicity (ACT), such as risk factors, monitoring indicators, and preventive measures, to provide possible direction for childhood cancer survivors suffering from cardiotoxicity.

## 2. Risk Factors

### 2.1. Treatment-Related Factors

The close link between the cumulative anthracycline dose and its cardiotoxicity risk is currently recognized; that is, the higher the cumulative dose, the higher the risk is [5]. A cross-sectional multivariable analysis of 1853 adult survivors of childhood cancer found that survivors exposed to 250 mg/m^2^ or more of anthracyclines had a higher incidence of cardiomyopathy than those who were not exposed [odds ratio, 2.7; 95% confidence interval (CI), 1.1–6.9] [6]. The International Late Effects of Childhood Cancer Guideline Harmonization Group developed a risk stratification system for cardiomyopathy to classify patients exposed to anthracycline at doses greater than 250 mg/m^2^ as high risk [7]. Modern chemotherapy regimens are constantly improving to keep drug doses in low-risk categories as much as possible. Unfortunately, cardiotoxicity also occurs in low-cumulative-dose populations, even below the tipping point [8]. In other words, there is no clear threshold for classifying safe anthracycline doses. It is worth mentioning that some adult survivors of childhood cancer have a long period of subclinical changes before they develop advanced cardiac adverse events [9]. In other words, cardiotoxicity is often silent early and does not have clinical manifestations until much later. This provides an opportunity for children with cancer exposed to high-dose doxorubicin to receive cardiac protection early and to avoid late irreversible heart damage.

Certain treatment combinations increase the risk of cardiotoxicity associated with anthracyclines, which is something that needs to be clarified before children with cancer receive related therapies. The Childhood Cancer Survivors Study reported that a significant number of childhood cancer survivors with a history of radiation have a high burden of cardiovascular diseases in early adulthood [10]. Childhood cancer survivors who received anthracycline chemotherapy and radiation, with or without irradiation in proximity to the myocardium, had an increased risk of cardiomyopathy. This cardiac damage is more powerful than that of anthracyclines or cardiac irradiation exposure alone [11]. Armstrong et al. evaluated the independent effect of chest-directed radiotherapy, without the confounding influence of anthracyclines, and found evidence of diastolic dysfunction in 22% of survivors exposed to radiotherapy alone [12]. The significant decrease in the left ventricular ejection fraction in children exposed to craniocerebral irradiation may be attributed to hypothalamus and pituitary dysfunction, leading to a decrease in the levels of growth hormones and insulin-like growth factor-1 [13]. This decrease suggests that growth hormone replacement therapy may be a new idea to prevent the development of cardiotoxicity. The current protocols, which comprise low but repetitive doses of radiation, have greatly reduced the direct tissue-damaging effects but have accelerated senescent changes in the cardiac conduction system that have led to an increased incidence of arrhythmia [14]. There are many debates regarding the cardiotoxicity effects of anthracyclines and other chemotherapeutic drugs. Preclinical and clinical research shows that high-dose cyclophosphamide, ifosfamide, fluorouracil, paclitaxel, trastuzumab, mitoxantrone, vincristine, and other drugs can induce cardiovascular system damage to varying degrees when used alone. Some drugs may not be statistically significant risk factors, but their combination does theoretically aggravate myocardial damage in those children [15]. There are few comparisons between single drugs and combination drugs for children with cancer because the combinations of anthracyclines and other chemotherapeutic agents have demonstrated excellent antitumor efficacy. Sledge et al. compared the effects of paclitaxel, doxorubicin, and the combination of paclitaxel and doxorubicin on cardiac function in breast cancer patients within a short period of time and demonstrated that paclitaxel monotherapy had a 3.7% incidence of cardiac complications compared to 8.7% after doxorubicin monotherapy and 8.6% after combined therapy with paclitaxel and doxorubicin [16]. Considering that anthracyclines are often used in combination with these potentially cardiotoxic drugs, the risk of combination administration should be considered before use to avoid underestimating cardiotoxicity.

Preclinical investigations suggest that decreasing the rate of infusion can reduce mitochondrial damage and thus decrease the probability of cardiac myocyte necrosis [17]. In order to achieve the long-term quality of survival without changing the individual dose, the investigators propose a modified method called “continuous infusion” to reduce the peak level of the concentration of doxorubicin in the blood by increasing the duration of the infusion but without reducing the dose [18]. This approach has been successful in short-term evaluations of adult cancer patients but not in children [19]. Lipshultz et al. compared short- and long-term effects between the two drug delivery methods in children with acute lymphocytic leukemia and found that cardiac protection and event-free survival did not differ between the two groups [20,21]. One explanation is that, even when the peak anthracycline serum concentrations are reduced, continuous infusion inevitably leads to prolonged cardiomyocyte exposure, which further aggravates myocardial injury. The multidisciplinary team reviewed clinical studies in adults and children, recommending the duration of anthracycline infusion be at least 1 h, although the precise optimal duration of extended infusion remains difficult to determine [22]. Even if the chosen infusion method does not achieve the ideal effect of short- or long-term weakening of cardiotoxicity, from a clinical perspective, long hospital stays, high costs, and adverse effects such as phlebitis are important factors limiting continuous infusion. In addition, liposome administration has been a new option in recent years. Liposomal anthracyclines have different pharmacokinetic properties, resulting in increased distribution of drug concentration in tumors and decreased concentration of free drugs in myocardial tissue. Therefore, liposomal anthracyclines have similar efficacy to the same dose of conventional anthracyclines but less cardiac toxicity (further discussed below).

### 2.2. Individual-Related Factors

Regardless of the drug dose level, the youngest children (less than 4 years of age at diagnosis) are particularly susceptible to anthracycline-mediated toxicity [5]. Völler and colleagues described a population-based pharmacokinetic model of doxorubicin in children that showed that infants and children aged < 3 years had a lower clearance of doxorubicin compared with older children and that the lower clearance was correlated with age [23]. The regenerative capacity of cardiomyocytes is limited. The application of anthracyclines during critical periods of organ development forced cardiomyocytes to undergo hypertrophy to maintain normal cardiac output once heart damage occurred. Chronic or late-onset heart failure is hard to avoid because of the inability of surviving myocytes to keep pace with normal body growth or other cardiac stress demands on the heart [24]. From this perspective, childhood cancer survivors face a higher heart risk than adults, and the younger they are, the greater is the risk.

Childhood cancer survivors have an increased risk of cardiovascular risk factors compared with normal children of the same age, and they are more likely to take hypotensor, hypoglycemic, and lipid-lowering drugs [25]. Childhood cancer survivors who received anthracycline chemotherapy had significantly increased rates of cardiac events and even death if they were accompanied by high blood pressure [26]. The interaction between specific cardiac events and cardiovascular risk factors such as hypertension, diabetes, dyslipidemia, and obesity, is not merely an additive effect but also provides positive feedback. Experts are calling on clinicians to conduct a baseline cardiovascular risk assessment in cancer patients scheduled to receive cardiotoxic cancer therapies [27]. Reviewing studies of childhood cancer survivors, we found that these risk factors would be equally applicable to children with cancer who are scheduled to receive anthracyclines. In addition, based on the collected standard risk factors, computer algorithms can be designed to build useful models to quantify the incidence of cardiotoxicity and to dynamically adjust it during long-term follow-up to better assess and develop improved prevention strategies in childhood cancer survivors. Practical models to predict heart failure onset before the age of 40 years for 5-year childhood cancer survivors (median age of 12 years) have been developed by Chow et al. Its secondary analysis, which explored the effects of obesity, hypertension, dyslipidemia, and diabetes in a subset of survivors with anthracycline exposure, found that cardiovascular risk factors have been shown to have little incremental information to prediction models of heart failure and ischemic heart disease as early as five years after diagnosis [28]. Although traditional cardiovascular risk factors have relatively low prevalence in young age, the incidence increases significantly after middle age (over 40 years of age) without any obvious plateau. Therefore, it is necessary to dynamically adjust risk factors during long-term follow-up, especially before middle age, to reduce the occurrence of serious cardiovascular events. Further studies are needed to clarify the usefulness and feasibility of predictive models for children with different age and disease types. Controlling traditional cardiovascular risk factors as a primary prevention strategy is expected to benefit children with cancer sooner than other heart protectants being explored. Abnormalities in left ventricular structure and function have been reported even in childhood cancer survivors who did not receive anthracyclines. Thus, the benefits of modulating conventional cardiovascular factors will extend far beyond survivors of ACT.

Girls with cancer seem more likely to achieve a clinical cure than boys; however, they appear to suffer from more severe cardiotoxicity [29]. In addition to pharmacokinetics, gender differences in cardiotoxicity have motivated a discussion of the effects of sex hormones on the heart. Left ventricular function and survival after administration of doxorubicin are significantly worse in knockout mice missing the androgen receptor than in the controls [30]. After further modeling the cells, part of the underlying mechanism for the protective effect of androgen on the heart was discovered, including the PI3K, AKT, and NOS-3 signaling pathways [31]. Preclinical studies have provided a mechanistic basis for the cardioprotective effects of estrogen, including increased mitochondrial biogenesis, protection from oxidative stress and apoptosis, stabilization of cardiac mast cells, promotion of favorable matrix remodeling, and pharmacokinetic differences [32]. The overall mixed conclusions suggest a gender predilection for cardiotoxicity in childhood cancer survivors because not all studies have found being female to be an unfavorable risk factor. Based on the differences in study end points, the evaluation methods, therapeutic doses, follow-up time, and the heterogeneity of the relationship between sex and cardiotoxicity need to be further clarified.

### 2.3. Genetic Polymorphisms

**Figure 1:**
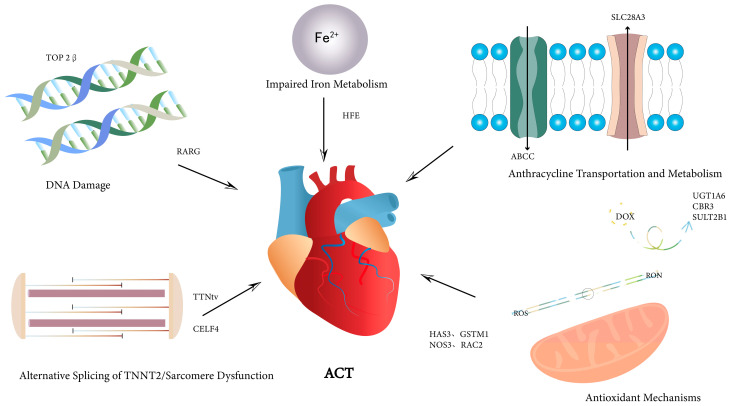
Summary of key affected genes with variants identified to be associated with anthracycline-related cardiotoxicity based on the proposed mechanisms of action. ACT, anthracycline-related cardiotoxicity; ABCC, adenosine triphosphate-binding cassette, subfamily C; CBR3, carbonyl reductase 3; CELF4, CUGBP, ELAV-like family member 4; DOX, doxorubicin; GSTM1, glutathione S-transferase M1; HAS3, hyaluronan synthase 3; HFE, hemochromatosis; NOS3, nitric oxide synthase 3; NOS, nitric oxide synthases; RAC2, ras-related C3 botulinum toxin substrate 2; ROS, reactive oxygen species; RARG, retinoic acid receptor gamma; SLC28A3, solute carrier family 28 member 3; SULT2B1, sulfotransferase family cytosolic member 2B1; TNNT2, cardiac troponin T; TOP2β, topoisomerase 2β; TTNtv, titin-truncating variant; UGT1A6, UDP-glucuronosyltransferase family 1A6.

Surprisingly, childhood cancer survivors who do not receive cardiotoxic therapy are at risk for heart abnormalities and increased systemic inflammation, but not all of those who have undergone toxic therapy experience cardiotoxicity. This observation opens up the possibility for genetic susceptibility. Aminkeng et al. searched the relationship between genetic factors and ACT and suggested that *RARG* rs2229774, *SLC28A3* rs7853758, and *UGT1A6* rs17863783 variants provide the strongest evidence of ACT in children [33]. Among them, *SLC28A3* rs7853758 is a protective gene that is negatively associated with the risk of cardiotoxicity and may impart more significant protection for children receiving higher doses of an anthracycline [34]. Recent studies demonstrated the cardioprotective effects of *SLC28A3* in patient-derived cardiomyocytes and identified a novel cardioprotective single nucleotide polymorphism, rs11140490, in the *SLC28A3* locus [35]. The mutation of *RARG* rs2229774 changed the function of inhibition of the Top2β by *RARG*, resulting in high Top2β expression, thereby inducing cardiotoxicity [36]. *UGT1A6* rs17863783, however, is thought to attenuate the *UGT1A6*-mediated glucuronidation process of anthracycline metabolites, leading to the accumulation of metabolites that can trigger cardiotoxicity [37]. A report from the Children’s Oncology Group suggested that the development of cardiomyopathy is associated with the myocardial accumulation of anthracycline metabolites. Homozygous disease of the G allele in *CBR3* led to an increased risk of anthracycline-associated cardiomyopathy in children with low to moderate doses (1–250 mg/m^2^), whereas no association was found in adults [38]. In addition, there have been anecdotal reports of hemochromatosis variants, the ATP-binding cassette transporter, cytochrome P450 oxidoreductase, remaining variants of solute carrier transporters, and NOS3 [39]. According to the mechanism of cardiotoxicity caused by anthracyclines, these genes are involved in many aspects of oxidative stress, iron loading, DNA damage, metabolism, sarcomere dysfunction, and so on [40]. The details are shown in Figure 1. Given the complexity of existing mechanisms, perhaps the search for genetic triggers of ACT should be broader. A successful example is the genome-wide association study by Professor Smita Bhatia and colleagues [41], who examined gene–anthracycline interactions and identified the *ROBO2* polymorphism as a susceptibility gene for anthracycline-associated cardiomyopathy. This discovery helps identify novel variants that are not known to be involved in the pathogenesis of ACT, that validate the relationship of ACT and the gene level and that promote the understanding of the pathogenesis [41]. Clearly, combining clinical and genetic risks is important for children who have been or will be exposed to anthracyclines and may in the future help predict cardiotoxicity and prevent potentially serious adverse events [42].

## 3. Screening Strategies

Although children with malignant cancers survive longer after chemotherapy as treatment options advance, more survivors are expected to be at risk of receiving ACT. The main research hotspots in the field of ACT include the role of doxorubicin in cardiotoxicity, the mechanisms, and the treatment strategies [43]. More studies are needed to explore the surveillance of clinical utility. Below we describe and review the advantages and limitations of relevant screening strategies to help clinicians make reasonable choices for patients.

### 3.1. Serum Biomarkers

Cardiac troponin has isoforms that are unique to cardiac myocytes, with higher sensitivity and superior tissue specificity and are associated with fewer false-positive results, which firmly established its advantages over other necrosis biomarkers in clinical studies as a good biomarker for detecting myocardial injury. Lipshultz et al. conducted a study in children receiving doxorubicin by collecting serum samples before, during, and after doxorubicin treatment and found that cTnT levels were elevated in 35% of the children after a median follow-up of 2.7 years [44]. This abnormality continued to occur in patients receiving low doses at an early stage [45]. Compared with large samples of the general population, we found that elevated levels of cTnT were rare in healthy subjects [46]. Therefore, any unexplained elevated cTnT level should indicate abnormality, and elevation in low-level cTnT represents subclinical cardiac damage. Highly sensitive cardiac troponin T (hs-cTnT) is more sensitive to myocardial injury than cTnT and has been validated in the community as a biomarker of cardiovascular risks, with changes significantly associated with subsequent heart failure [47]. It is currently believed that hs-cTnT, a valuable marker of myocardial injury, is more significant in early-onset or acute cardiotoxicity than late heart damage and makes a significant contribution to monitoring the response to cardioprotective treatment [48]. In the updated definition of asymptomatic cancer therapeutics-related cardiac dysfunction induced by anthracyclines, cardiac biomarkers including troponin have been initially included as important references for evaluation [49]. However, it is important to emphasize that the most important limitation of hs-cTnT application in childhood is the lack of specific reference intervals for age groups, which severely affects the collection of clinical data [50]. Despite the different analytical properties of hs-cTnI and hs-cTnT, the biomarker variations between samples expressed as percentages are similar. All guidelines about the general population basically agree that both methods have similar diagnostic accuracy and can be used to diagnose an acute or chronic myocardial injury [51].

Small-scale anthracycline-related clinical trials in childhood cancer survivors support the identification of individuals at risk of serious heart injury by N-terminal pro-B-type natriuretic peptide (NT-proBNP) [52]. A report from the St. Jude Lifetime Cohort reported that survivors exposed to the highest doses of anthracycline (dose > 350 mg/m^2^), compared to no anthracycline, had a threefold increase in risk for abnormal NT-proBNP (risk ratio, 2.99; 95% CI, 2.27–3.95) [53]. NT-proBNP may be a valid indicator, but considering that inflammation may be a trigger for its release, the interference of inflammatory conditions should be interpreted with caution when studying NT-proBNP levels [54]. On the one hand, the interfering factors for NT-proBNP have limited its utility as a reliable screening marker for subclinical cardiotoxicity in children. On the other hand, the lack of correlation between serological markers and left ventricular ejection fraction (LVEF) has become increasingly apparent in the study of cardiotoxicity, such as cardiac troponin and NT-proBNP [55,56]. Identifying the relationship between biomarkers and LVEF may not be as accurate as studying the relationship with clinical symptoms because LVEF also changes dynamically during short-term follow-up visits. Isolated elevations in natriuretic peptides without validated imaging parameters may be considered as biochemical evidence of cardiotoxicity but cannot be relied upon alone as a basis for decisions regarding cancer treatment continuation versus discontinuation [49].

### 3.2. Conventional and Novel Electrocardiography Methods

A meta-analysis evaluated electrocardiograph (ECG) abnormalities in childhood cancer survivors after cardiotoxic therapy. Multiple ECG abnormalities were described, and they may or may not be meaningful [57]. A retrospective study found that a prolonged QTc interval was associated with subsequent left ventricular dysfunction in childhood cancer survivors treated with anthracyclines [58]. An ECG seems to be the most commonly used screening method, with the advantage of cheapness and availability, but electrolyte disturbance and the application of combinations of drugs play a certain interference role. Frequent false-positive results lead to delays in chemotherapy and additional examinations. In response to this problem, some scholars propose providing feedback to the electrophysiological changes in ECGs via dynamic learning, which provides earlier information through dynamic feature extraction to predict the occurrence of adverse events, so as to achieve early detection and early prevention [59]. A cardiodynamicsgram is a visual representation of the internal dynamic pattern of ECG signals obtained via dynamic learning, which can sensitively capture small ischemic changes implied by the electrocardiogram and can be used as a new supplementary diagnostic tool for monitoring subclinical cardiotoxicity. This assay is simple and noninvasive, providing convenient conditions for prospective clinical studies and may be an important way to monitor anthracycline cardiotoxicity in the future.

### 3.3. Echocardiography and Multimodality Imaging

For children at intermediate to high risk receiving potentially cardiotoxic chemotherapy, the current practice guideline recommends routine echocardiographic screening at baseline, during anthracycline therapy, and after completion of treatment for early detection of subclinical cardiac dysfunction. The guideline recommends surveillance echocardiograms at 2-year intervals for survivors at high risk and 5-year intervals for survivors at moderate risk, but it no longer recommends screening survivors at low risk (strong recommendation) [60]. Left ventricular fractional shortening and LVEF are the most commonly used measurements in children. In fact, if there is a decrease in left ventricular fractional shortening and LVEF, heart involvement is more than just an early or mild sign. The St. Jude Lifelong Cohort Study found evidence of cardiac dysfunction in one-third of survivors with normal LVEF in the analysis of the prevalence of cardiac insufficiency in adult survivors of childhood malignancies. They reported that abnormal global longitudinal strain (GLS), which reflects early myocardial alterations, was more prevalent than LVEF reduction and was more closely associated with children exposed to high cumulative doses of anthracycline [12]. Gonzalez-Manzanares et al. first used automated software to analyze the role of GLS in screening for ACT in a study of survivors of childhood leukemia and found a higher prevalence of left ventricular systolic dysfunction detected using GLS than with LVEF among patients with long-term GLS (26.6% vs. 12.2%) [61]. Given that abnormal LVEF and GLS are not equally distributed, it is reasonable to assume that they together constitute complementary measures of systolic dysfunction among long-term childhood cancer survivors [62]. Furthermore, diastolic dysfunction should be suspected when the patient presents with symptoms and signs of heart failure but ventricular systolic function is normal or near-normal. A prospective study of child survivors found a positive association between anthracycline doses and diastolic dysfunction [63]. Comprehensive echocardiographic surveillance of patients with pediatric cancer should include serial assessment of right ventricular (RV) size, RV function, and RV systolic pressure [64]. Exercise echocardiography can provide some monitoring for diastolic abnormalities [65].

Compared with echocardiography, serial resting radionuclide angiocardiography has the advantages of high accuracy and low subjective variability [66]. Lack of detailed information regarding cardiac structure and diastolic function limits its application as a primary surveillance modality in cancer survivors. In contrast, cardiac magnetic resonance imaging (CMRI), although also expensive and not readily available, does not require ionizing radiation exposure and may provide additional information about the cardiac structure and function [67]. Currently, only limited data on the use of CMRI in childhood cancer patients are available. For clinical purposes, current recommendations are to consider the use of CMRI as a follow-up study for technically inadequate echocardiography (moderate recommendation) [68]. Abnormal myocardial characteristics and strain parameters in CMRI are thought to quantify diffuse fibrosis in children with normal ejection fraction. This technology will be considered as an alternative method for subclinical cardiotoxicity judged via echocardiography [64].

Studies have shown that decreased LVEF does not always predict symptomatic events such as heart failure or myocardiopathy in children, and biomarkers rooted in dynamic monitoring have not yet been proven to be surrogate end points for cardiovascular disease. Screening for a single indicator during chemotherapy rarely identifies significant cardiac damage that changes treatment decisions, and it is difficult to reflect true cardiotoxicity or predict heart damage years later. Therefore, a combination of serological and imaging modalities may help to monitor the anthracycline-induced cardiovascular changes [7]. The limited screening strategies lead us to deem that a simple screening test to confirm the presence of subtle abnormalities is necessary for a comprehensive evaluation at a later stage. 

## 4. Prevention

A randomized controlled trial in children with acute lymphocytic leukemia showed that increased cTnT levels during the first 90 days of doxorubicin therapy were associated with lower LV mass and LV end-diastolic posterior wall thickness after 4 years (*p* < 0.01) [69]. This result suggests that the first 90 days of anticancer therapy may be a reasonable window for the use of cardioprotective agents. Although there are no large-scale clinical trials to verify this suggestion, the fact that some people still develop progressive disease after the intervention warns us that it is desirable to intervene at an early point in time, especially because the efficacy of existing cardioprotective drugs remains to be explored in more detail (Table 1).

### 4.1. Cardioprotective Drugs

Dexrazoxane is the therapy approved by the United States Food and Drug Administration for the prevention of ACT. It inhibits the binding of anthracyclines to topoisomerase IIβ and is not limited to the redox reaction of iron-chelating agents, thus reducing cardiotoxicity [70]. Dexrazoxane should be effective in reducing cardiac event-free survival in pediatric patients with high cumulative anthracyclines, while not affecting the antitumor effects of chemotherapy drugs [71,72]. Most researchers support using dexrazoxane, but the biggest concern is the occurrence of a secondary tumor, especially in children with prolonged survival [73]. The failure to meet statistical criteria and the interference with other drugs made the Pediatric Oncology Group’s report controversial. In particular, recent studies did not observe the development of secondary tumors in late health outcomes after treatment with dexrazoxane [74,75]. The alarming potential risk should be a wake-up call, requiring clinicians to carefully weigh the risks and benefits. After a comprehensive evaluation, the International Late Effects of Childhood Cancer Guideline Harmonization Group proposed that the benefit of the treatment may outweigh the risk of subsequent tumors when the cumulative doxorubicin or equivalent dose is at least 250 mg/m^2^ (moderate recommendation) [76]. A recently published prospective multicenter study has provided the encouraging news that, compared with long-term survivors of childhood tumors who did not receive dexrazoxane treatment, a reduced risk of having lower left ventricular function (fractional shortening < 30% or ejection fraction < 50%; odds ratio, 0.24; 95% CI, 0.07–0.81) was reflected in those who did receive it, even after 20 years, although this protective effect was seen mainly in children with a cumulative doxorubicin dose ≥ 250 mg/m^2^ [77]. Current clinical practice does not support the use of dexrazoxane in all populations [78]. However, according to previous experience, even low doses of doxorubicin showed unexpected cardiotoxicity; so, the issue of how we can better realize the shift from low-risk to risk-free populations needs further research to make breakthroughs in the most promising drug.

Doxorubicin can be embedded by small molecules of liposome and extravasated through the tumor vasculature with increased permeability, resulting in an increased concentration of doxorubicin in tumor tissue. This selective drug delivery method increases the therapeutic effect of liposomal doxorubicin and reduces the occurrence of heart damage [79]. On the other hand, embedding results in the slow release of doxorubicin, which reduces the peak plasma concentration of doxorubicin and reduces acute cardiotoxicity. PEGylated liposomal doxorubicin or lipoplex is reported in adults to exhibit similar clinical efficacy, less cardiotoxicity, and greater safety [80]. One study compared liposomal daunorubicin with idarubicin in children with acute myelogenous leukemia, showing that the drug had a good overall profile in the incidence of acute and advanced cardiotoxicity and eventually allowed an increased anthracycline dose without increasing cardiotoxicity [81]. In children, there are few clinical studies with liposomal doxorubicin. The modest gap led us to find that children treated with liposomal doxorubicin are also at risk of cardiotoxicity and other serious toxicities, such as anaphylaxis, mucositis, infection, and hepatorenal toxicity. It is unclear whether the exact cumulative incidence and the risk are lower than those of conventional anthracyclines [82]. Although these liposomes have the clear advantage of precise localization, they may still leak a fraction of doxorubicin into the plasma, which explains the clinical toxicity. The data suggest that liposomal anthracyclines, particularly PEGylated liposomal doxorubicin, will provide clinical benefit when the risk of ACT is higher.

Neurohormonal-blocking drugs had been preliminarily used in clinical trials in children exposed to anthracyclines, but insufficient research and mixed results have limited roll-out and cannot be recommended for routine use. Systematic reviews of randomized controlled trials found that the short-term cardioprotective effect of the angiotensin-converting enzyme inhibitor (ACEI), primarily enalapril, was observed in terms of LVEF, cTnI, CK-MB, and NT-proBNP levels [83] and did not affect the malignancy response rate or the risk of death [84]. Silber et al. carefully designed a prospective study of ACEI intervention in long-term survivors of childhood cancer exposed to anthracyclines and found that during the first year of treatment, left ventricular end-systolic wall stress changed at a higher rate in the enalapril group than in the placebo group, but over time, long-term studies have shown that the protective effect diminishes and the heart wall becomes thinner [85]. Enalapril, a promising cardiac protector, has been associated with dizziness or hypotension (risk ratio, 7.17; 95% CI, 1.71–30.17; *p* = 0.007) and a higher risk of fatigue (Fisher’s exact test, *p* = 0.013) in children [86]. Clinicians should weigh the possible benefits of enalapril against known side effects in survivors of childhood cancer with ACT without symptoms. Compared to ACEI, angiotensin receptor blockers or aldosterone antagonists also act on the renin–angiotensin–aldosterone system, showing the presence of signals of preventing deterioration of systolic and diastolic function as well as of attenuating cardiac fibrosis and myocyte apoptosis caused by anthracycline in patients with breast cancer. But they are used much less frequently in children, making it difficult to infer cardiac protection. Carvedilol is the best beta-blocker for ACT, being the most used and the most effective, followed by metoprolol [87]. This result is not only attributed to beta-blockers antagonizing catecholamines but probably also to carvedilol’s unique antioxidant properties [88]. Drawing on successful experience in adult cancer patients, an RCT of children treated with doxorubicin reported that carvedilol significantly reduced left ventricular systolic dysfunction, as reflected by improvement in fractional shortening and global peak-systolic strain on two-dimensional echocardiography [89]. Current research results are promising, and larger, longer-term clinical studies are needed to demonstrate the true efficacy of these drugs, especially in children with cancer who are receiving or have received doxorubicin therapy. An ongoing clinical trial involving pediatric cancer survivors treated with high-dose anthracyclines discusses the effect of low-dose carvedilol in preventing heart failure in this population (NCT02717507). Whether pharmacological reversal of ventricular remodeling can be achieved in childhood cancer survivors at risk for heart failure is of concern.

In animal studies, statins have shown no less protective potential than carvedilol through anti-inflammatory and antioxidant effects [90]. A current trial of statins for preventing ACT randomly assigned 300 newly diagnosed adult patients with lymphoma to atorvastatin or a placebo and is expected to assess its authenticity for cardiac protective function via CMRI and echocardiography 2 years after anthracycline treatment (NCT02943590). Childhood cancer survivors treated with anthracyclines are at higher risk of developing metabolic syndrome [91]. Long-term application of statins may theoretically have a cardioprotective effect on patients with several cardiovascular risk factors, but more high-quality cohort studies need to be conducted and generalized to children. Epirubicin, daunorubicin, mitoxantrone, and other doxorubicin substitutes need to be repeatedly confirmed for their relationship between cardiotoxicity and antitumor efficacy [92]. Antioxidants such as the vitamin family, coenzyme Q10, glutathione and L-carnitine, and dietary supplements such as N-acetylcysteine are promising and are currently transitioning from preclinical studies to small clinical trials [93,94,95,96]. All of the studies have methodological limitations, and conflicting results require more clinical data than just animal studies. Flavonoids and other phytochemicals [97] and hematopoietic stimulating factors [98,99,100] are being explored in basic experiments, which may provide cheap and effective solutions for the early prevention of cardiotoxicity.

### 4.2. Lifestyle

In addition to drug intervention, it is worth mentioning the effect of exercise on cardiotoxicity. Anthracyclines can cause left ventricular dysfunction by apoptotic cardiomyocytes. Correspondingly, aerobic training is beneficial for improving myocardial contractility, increasing cardiac output, even reversing ventricular remodeling [101]. A meta-analysis included nine clinical studies of children who were cancer survivors receiving cardiotoxic agents that demonstrated that aerobic exercise has a statistically and clinically significant positive effect on cardiorespiratory fitness (effect estimate = 6.92%, *p* value = 0.02) [102]. A second roundtable was held in 2018, and experts stated that the ability of exercise to prevent or improve cardiotoxicity is still poorly documented but that the clinical and subclinical trends point in the direction of exercise cardio-oncology [103]. Delayed factors may interfere with the correlation between heart function and exercise, such as sedentary lifestyle and psychosocial factors, which to some extent indicate the complicacy of standardizing aerobic exercise to prevent cardiac involvement. The type of exercise that does not aggravate the heart load and the safely tolerated dose are factors that need to be considered in clinical situations. Survivors can be encouraged to exercise regularly, as appropriate, under close monitoring, and to determine appropriate safe tolerances based on the risk of cardiotoxicity through rational grading [104]. Similar to the tertiary prevention of tumors, childhood survivors who took anthracyclines can receive early non-drug treatment under the guidance of physicians, promote cardiorespiratory health, and reduce the cumulative risk of cardiotoxicity. If high-risk groups can be screened through the risk assessment model as soon as possible, if cardiotoxicity can be properly monitored, and if cardioprotective drugs can be used in preventive ways, the clinical results may meet the needs of most children.

### 4.3. Genetic Screening

A key determinant of wide range of inter-individual susceptibility to anthracycline-related cardiotoxicity is the underlying genomic variation in individual patients. Various genetic variants associated with ACT, such as SLC28A3 rs7853758, RARG rs2229774, and UGT1A6 rs17863783, have been shown to improve risk stratification in children with cancer [105]. Thus, genetic testing may support childhood cancer patient care decisions to improve prevention of ACT. Pediatric facilities across Canada have evaluated the practicality and economic benefits of pharmacogenetic testing as a tool. In their study, this genetic screening could reduce mortality by approximately 17% and could reduce costs by about 6%, which undoubtedly provides a feasible new way to reduce the heart damage caused by anthracyclines [106]. Genetic screening, which divides children at low and high risk of cardiotoxicity, not only helps prevent heart damage but also guides subsequent treatment. For children in the low-risk group, they will be likely to receive longer durations and higher doses of doxorubicin, which are conducive to adjusting the chemotherapy regimen and improving the chemotherapy effects. However, for children in the high-risk group, they are more suited for the administration of cardioprotective drugs than those in the low-risk group. In addition, if the association of other genetic variants with ACT is validated in subsequent studies, it would be reasonable for them to be included in a clinical practice guideline as part of clinical risk predictors.

## 5. Conclusions

Pediatric cardio-oncology is helping clinicians to identify and protect against ACT. Risk factors, including genetics, promise to be a cheap and low-risk tool for predicting cardiotoxicity and will become a priority with the universal use of anthracyclines, the aging of childhood cancer survivors, and the intensive occurrence of cardiovascular damage. Monitoring subclinical cardiotoxicity is an important component of the prevention of subsequent overt heart damage. The promising indicators may predict cardiac deterioration, which requires long-term monitoring to verify. Exploring simple screening methods or a combination of multiple tests in high-risk groups may be clinically effective strategies. Protecting the heart at an earlier age is the critical task when treating high-risk children; otherwise, a heart transplant would be the last line of defense for them. Although dexrazoxane is currently considered the most promising cardioprotective agent, the best preventive strategy still needs to be adjusted by randomized clinical studies with long-term follow-up. We appeal to interested oncologists, cardiologists, epidemiologists, geneticists, nurses, and others to join together in a multidisciplinary alliance to help improve the quality of life for childhood cancer survivors.

## Figures and Tables

**Table 1:** Summary of the drugs used to prevent cardiotoxicity.

Type	Drug	Mechanism	Study Subject	Challenges
Cardioprotective agent	Dexrazoxane	Iron-chelating agent	Human	Dispute of long-term efficacy and untoward effects
Liposome	Liposomal anthracycline, PEGylated liposomal doxorubicin	Different drug distribution	Human	Minimal research on pediatric data
Neurohormonal antagonists	ACEI, ARB, beta-blocker,	Neurohormonal antagonists	Human	Limited study quality Lack of clinical trials
	aldosterone antagonist, renin-inhibitor		Animal	
Statins	Atorvastatin	Antioxidative and anti-inflammatory effects	Human	Minimal research on pediatric data
Anthracycline derivatives	Epirubicin, daunorubicin, mitoxantrone	Equivalent replacement	Human	Conflicting information
Dietary supplements	Vitamins A, B, C, E, glutathione, selenium, l-carnitine,	Antioxidant	Human	Limited efficacy
	coenzyme Q_10_, *N*-acetylcysteine	Mucolytic agent	Human	Limited efficacy
Plant-based treatment	Flavonoids, apigenin, quercetin, pinocembrin	Ongoing	Animal	Lack of clinical trials
Hematopoietic stimulator	TPO EPO G-CSF	Ongoing	Animal	Lack of clinical trials

ACEI, angiotensin-converting enzyme inhibitor; ARB, angiotensin receptor blockers; EPO, erythropoietin; G-CSF, granulocyte colony-stimulating factor; TPO, thrombopoietin.
